# The N-Terminal Region of Nurr1 (a.a 1–31) Is Essential for Its Efficient Degradation by the Ubiquitin Proteasome Pathway

**DOI:** 10.1371/journal.pone.0055999

**Published:** 2013-02-07

**Authors:** Beatriz Alvarez-Castelao, Fernando Losada, Patrícia Ahicart, Jose G. Castaño

**Affiliations:** Departamento de Bioquímica, Instituto de Investigaciones Biomédicas “Alberto Sols”, Universidad Autônoma de Madrid-Consejo Superior de Investigaciones Científicas, Centro de Investigación Biomédica en Red sobre Enfermedades Neurodegenerativas, Facultad de Medicina Universidad Autônoma de Madrid, Madrid, Spain; CNRS UMR7275, France

## Abstract

NURR1/NR4A2 is an orphan nuclear receptor that is critical for the development and maintenance of mesencephalic dopaminergic neurons and regulates transcription of genes involved in the function of dopaminergic neurons directly via specific NGFI-B response elements (NBRE).and substantial data support a possible role of Nurr1 in the pathogenesis of Parkinson's disease (PD). Here we show that Nurr1 is degraded by the ubiquitin-proteasome pathway and determined that N-terminal region (a.a 1–31) of Nurr1 is essential for an efficient targeting of Nurr1 to degradation in the cell. Nurr1 Δ1–31 has a much longer half-life, and as a consequence its steady-state protein levels were higher, than full-length Nurr1 in the cell. Nurr1 Δ1–31 was as potent as Nurr1 full length in transcriptional luciferase reporter assays after normalization with the corresponding steady-state protein expression levels, either in trans-activation of NBRE or trans-repression of iNOS (inducible NO synthase) reporters. These results suggest that Nurr1 Δ1–31, because of longer persistence in the cell, can be a good candidate for gene and cell therapies in the treatment of PD.

## Introduction

NURR1 (Nur-related factor 1, NR4A2/NOT1/RNR-1/HZF-3/TINUR) gene, a member of nuclear receptor superfamily [1], [2], is an orphan nuclear receptor that behaves as a transcriptional activator in the central nervous system, and is required for the development of mesencephalic dopamine (mesDA) neurons. It is highly expressed in mesDA neurons during development and throughout adulthood [3] [4] [5]. In mice lacking NURR1, mesencephalic precursors fail to undergo terminal differentiation and adopt a mature dopaminergic phenotype, dying as development progresses [6], [7], [8]. Nurr1 is implicated in the differentiation, survival, connectivity and migration of mesDA neurons. Importantly, Nurr1 directly induces transcription of tyrosine hydroxylase, the rate limiting enzyme in the synthesis of dopamine [9] [10] [11], as well as other important dopaminergic markers, including the dopamine transporter and vesicular monoamine transporter 2 [12]. Nurr1 is also required for maintenance of maturing and adult dopaminergic neurons [13]. Nurr1 contains N- and C-terminal activation domains (AF-1 and AF-2, respectively) thought to regulate its transcriptional activity [14], [15], [16], [17]. Nurr1 transcriptional activity is positively regulated by mitogen-activated protein kinase (ERK1/2, ERK5) signalling via the N-terminal AF-1 region, and ERK1,2/ERK5 phosphorylation sites have been identified proximal to the AF-1 core of Nurr1 [18] [16] [19] [20] [21] and negatively regulated by LIMK1 [21]. Nurr1 also functions as a trans-repressor of pro-inflammatory gene promoters in macrophages, microglia and astrocytes by recruiting CoREST corepressor complex [22].

The important role that Nurr1 plays in dopaminergic neurons has been underscored by the identification of several changes in its gene that are associated with Parkinson's disease (PD). Two monoallelic mutations in the 5′ region of Nurr1 gene (c.-291delinsT and c.-245T>G) have been shown to be associated with PD, those mutations reduced the expression of Nurr1 [23], a homozygous 7048G7049 polymorphism was found in intron 6 of the Nurr1 gene in association with PD [24], a missense mutation (S125C) in Nurr1 has been described in a PD patient [25] and a single base substitution in the 5′-UTR (c.-309C>T) correlated with a decrease in Nurr1 mRNA expression has also been described in PD patients [26]. Furthermore, Nurr1 expression is reduced in neurons with pathological signs in brains of PD patients [27] and a decrease in Nurr1 activity is observed in peripheral blood lymphocytes of PD patients [28]. As a consequence, it has been suggested that Nurr1 can be a potential target to develop novel therapeutic strategies in PD aimed to enhance the survival of mesDA neurons to stress [15], [29], [30].

We have approached the study of the degradation pathway of Nurr1 because its importance in the maintenance of the dopaminergic phenotype, its implication in PD and its role as a protector for adult dopaminergic neurons. Previously it has been shown that Nurr1 is degraded by the ubiquitin proteasome pathway [31]. Here we confirmed that Nurr1 is degraded by the proteasome pathway and this degradation is dependent of the N-terminal region of Nurr1 (aminoacids 1 to 31). Deletion of this N-terminal region of Nurr1 produce a rather stable Nurr1 protein with full capabilities as transcription factor, accordingly Nurr1 Δ1–31 construct could be an excellent candidate for its use in genetic and cell therapeutic strategies for PD patients.

## Materials and Methods

### Recombinant DNA constructs

DNA constructs for expression of mouse Nurr1 and Flag-Nurr1 were generated from a mouse Nurr1 cDNAs and cloned into pcDNA3.1 (Zeo+) either untagged or Flag-tagged in the N-terminus. The construct Nurr1 1–337 was produced by PCR introducing a stop codon at position 338 of mouse Nurr1 sequence using the following oligonucleotides: 5′Nurr1 1–337 5′-CGCACGGACAGTTAAAAAGGCCGGAGAGG-3′ and 3′Nurr1 1–337 5′-CCTCTCCGGCCTTTTTAACTGTCCGTGCG-3′. The constructs Nurr1 Δ163–187, Nurr1 Δ163–217 and Nurr1 Δ163–247 internal deletion mutants were obtained by PCR from mouse Nurr1 construct in pcDNA 3.1 by substituting the NdeI/XhoI cassette of Nurr1 (NdeI cleaves after nucleotide 481, leaving in aminoacids 1–162) with the products of amplification (digested with NdeI/XhoI) of mouse Nurr1 obtained with the following oligonucleotides:

5′NdeI-Nurr1 Δ163–187 5′-GACGCATATGTCTAGCTGCCAGATGCGCTTCGAC-3′, 5′NdeI-Nurr1 Δ163–217 5′-GCGACATATGTTCGCCGTGCCCAACCC-3′, 5′NdeI-Nurr1 Δ163–247 5′-GCACGCATATGTCGCAGTTGCTTGACAC-3′ and a common reverse primer 3′XhoI-Nurr1 5′-AGCGCTCGAGTTAGAAAGGTAAGGTGTCCAGG-3′. The constructs Nurr1 Δ1–96, Nurr1 Δ1–161 and Nurr1 Δ1–262 N-terminal deletion mutants were obtained from mouse Nurr1 construct in pcDNA3.1 and the following oligonucleotides: 5′Nurr1 Δ1–96 5′-CGAAAGCTTATGCACAATACCAGCAACACAGCC-3′, 5′Nurr1 Δ1–161 5′-CCGAAGCTTATGATCGAGGCAGAGGAAGAC-3′, 5′Nurr1 Δ1–262 5′-GGCAAGCTTATGTGCGCTGTTTGCGGTGACAACG-3′ and the common reverse primer 3′XhoI-Nurr1. The constructs Nurr1 Δ1–80, Nurr1 Δ1–63, Nurr1 Δ1–43 and Nurr1 Δ1–31, N-terminal deletion mutants were obtained by PCR from mouse Nurr1 construct in pcDNA3.1 with the following oligonucleotides: 5′Nurr1 Δ1–80 5′-GCGTAGATCTATGCCCCTGTCCGGACAGC-3′, 5′Nurr1 Δ1–63 5′-GCGTAGATCTATGGACAACTACAGC-3′, ′Nurr1 Δ1–43 5′-GCGTAGATCTATGGACCTCACCAAC-3′, 5′Nurr1 Δ1–31 5′-GCCTAGATCTATGGATTTCTTAACTCC-3′ and the common reverse primer 3′XhoI-Nurr1. Introduction of the point mutation S125C into Nurr1 coding sequence was obtained by site-directed mutagenesis using the Stratagene “Quick-base change” method. Nurr1 triple Pro/Ala mutant (Nurr1 P2/12/17 A) was obtained by amplification from wild type Nurr1 with the following primers:: 5′Nurr1 P2/12/17A: 5′-GCGTAGATCTATGGCTTGTGTTCAGGCGCAGTATGGGTCCTCGGCTCAAGGAGCCAGCGCCGCTTCTCAGAGC-3′ and 3′ Xho reverse primer: 5′-AGCGCTCGAGTTAGAAAGGTAAGGTGTCCAGG-3′, fragment was digested with BglII/XhoI and ligated into pcDNA3.1 (Zeo+) digested with the same restriction enzymes. All constructs were completely sequenced by automatic DNA sequencing

### Study of endogenous Nurr1 degradation in PC12 cells

Rat PC12 cells were grown in Dulbecco's modified Eagle's medium (DMEM, Gibco BRL) supplemented with 10% horse serum (Gibco BRL), 5% foetal bovine serum (Gibco BRL) and 100 µg/mL gentamycin, at 37°C and 5% CO_2_ in P60 Petri dishes. Cells were treated with 25 µg/ml cycloheximide (CHX) in the absence or in the presence of 10 µM lactacystin for the times indicated up to 12 h. After the treatments, cells were washed in phosphate-buffered saline (PBS) and lysed in 200 µl of lysis buffer per well plate (50 mM Tris-HCl pH 7.5, 150 mM NaCl, 1% NP40, 2% SDS, 1% deoxycholate, 20 µM leupeptin, 10 µg/ml pepstatin, 1 mM PMSF); Cell extracts were sonicated for 10 min on ice, centrifuged at 14000×g for 30 min at 4°C and the supernatants were used to measure total protein concentration by BCA protein assay kit (Thermo Scientific-Pierce). Total proteins (50 µg) were separated onto 10% SDS-PAGE gels and transferred to nitrocellulose membrane for Western immunoblot analysis. Membranes were blocked with TTBS (50 mM Tris-HCl pH 7.5, 150 mM NaCl, 0.1% Tween-20) with 3% BSA o.n. The blots were then probed with anti-Nurr1 antibodies 1∶1000 (Santa Cruz Biotechnology, Sc-990 or Sc-991 directed against the C-terminal and N-terminal region of Nurr1, respectively) and anti-tubulin (1∶1000, DM1A, Sigma) as loading control. Signals from the primary antibodies were amplified using species-specific antibodies to rabbit or mouse IgG conjugated with horseradish peroxidase (Bio-Rad). Immunoblots were developed by direct capture of chemiluminescence with DNR MF-ChemiBIS 3.2 Bio-Imaging System and quantification with Totallab TL100 software. Results are expressed as mean ± s.e.m. for a minimal number of three independent experiments.

### Studies of ectopically expressed Nurr1 degradation in HeLa cells

HeLa cells were grown in Dulbecco's modified Eagle's medium (DMEM, Gibco BRL) supplemented with 10% foetal bovine serum (Sigma-Aldrich) and 100 µg/mL gentamycin, at 37°C and 5% CO_2_. HeLa cells were plated at 3×10^5^cells/well in 6-well plates and transfected with Lipofectamine (Invitrogen). Transfected cells were treated with the protein synthesis inhibitor CHX (25 µg/ml) for the times indicated; 10 µM Lactacystin, or 50 nM Leptomycin B was added where indicated. Transfected cells were processed as described above for PC12 cells and analyzed by immunoblot using the following primary antibodies: anti-Nurr1 (1∶1000, Santa Cruz Biotechnology Sc-990 and Sc-991), or anti-tubulin antibodies (1∶1000, Sigma, DM1A) as control for protein loading. Values reported are means ± s.e.m. from three independent experiments.

For the study of Nurr-1 ubiquitylation, cells were co-transfected with HA-tagged ubiquitin construct (provided by Dr. Dirk Bohmann, Department of Biomedical Genetics, University of Rochester Medical Center, Rochester, USA).plus Nurr1 full-length or Nurr1 Δ1–31 deletion construct. Transfected cells (36 h after transfection) were incubated in the presence or in the absence of 10 µM lactacystin for 12 h. Cells were lysed in 50 mM Tris-HCl pH 7.5, 150 mM NaCl, 0.5% NP40, 20 µM leupeptin, 10 µg/ml pepstatin, 1 mM PMSF and the clear lysates were immunoprecipitated with anti HA-antibody (Roche) previously coupled to protein G-Sepharose (GE-HealthCare). The immunoprecipitates were loaded onto 10% SDS-PAGE, transferred to nitrocellulose, and developed with anti-Nurr1 antibody (1∶1000, Sc-990, Santa Cruz Biotechnology).

To study Nurr1 mediated transactivation, HeLa cells were transfected with pcDNA Nurr1 or pcDNA Nurr1 Δ1–31 and Δ1–80, the reporter plasmid 3xNBRE-tk-Luc containing 3 copies of the Nurr1-responsive element (provided by Dr. Thomas Perlmann, Ludwig Institute for Cancer Research Ltd., Stockholm, Sweden) and *Renilla* as a reporter control. Luciferase activity was assayed with the Dual-Luciferase reporter assay system from Promega according to the manufacturer's instructions. For the studies of Nurr-1 trans-repression, RAW 264.7 macrophage cell line was grown in RPMI plus 15% fetal bovine serum and transfected by nucleofection (Nucleofector, Lonza AG) as per manufacturer's protocol with pcDNA (mock), pcDNA Nurr1 or pcDNA Nurr1 Δ1–31, the reporter plasmid iNOS (inducible NO synthase) murine-luciferase piNOSm-luc [32] provided by Dr. Manuel Fresno, Centro de Biología Molecular Severo Ochoa, Madrid, Spain) and Renilla as reporter control. Transfected RAW 264.7 cells were stimulated with bacterial lipopolysaccharide (LPS, Sigma) at 0.1 µg/ml for 8 h and luciferase assays were performed as described above. For both type of transcriptional assays results are expressed as means ± s.e.m. for the quotient of the activities of firefly and *Renilla* luciferase.

### Immunofluorescence and confocal microscopy

Cells were grown on coverslips and 36 h post-transfection coverslips were washed 3 times with cold PBS, fixed with 4% paraformaldehyde in PBS for 20 min and permeabilized and blocked with PBS, 1% Triton X-100 containing 3% BSA for 1 h at room temperature. Primary anti-Nurr1 antibody (1∶1000, Santa Cruz Biotechnology Sc-990) was added in the blocking solution without Triton X-100, and incubated for 2 h at room temperature. After washing 5 times (each for 5 min) with PBS, coverslips were incubated with Alexa Fluor 488 conjugated anti-mouse antibody (1∶500 dilution) for 1 h, washed again 5 times with PBS. For nuclear visualization 4′,6-diamidino-2-phenylindole (DAPI, 5 µg/ml) was added in the first wash with PBS. Coverslips were finally mounted with ProLong for confocal microscopy observation in a laser scanning microscope (Carl Zeiss LSM-510). Images were captured with the same settings for each set of experiments. Controls, omission of primary or secondary antibodies, revealed no fluorescence.

## Results

### Proteasomal degradation of Nurr1

**Figure 1:**
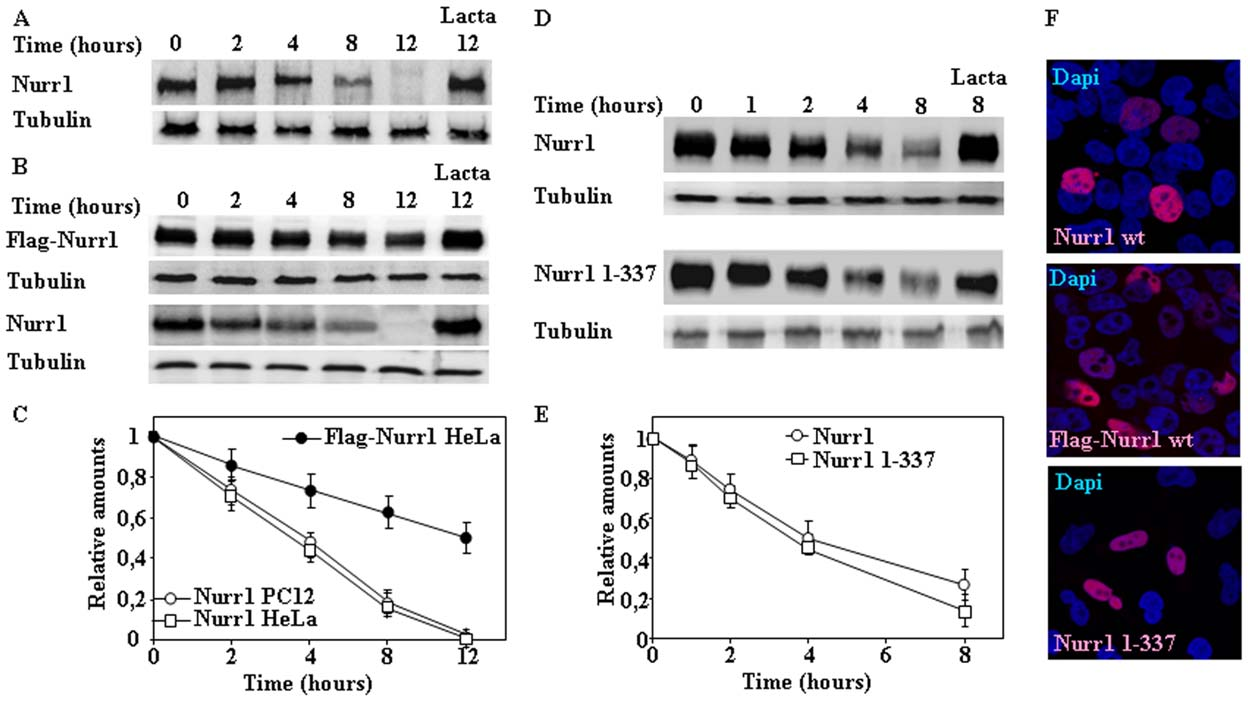
Degradation of endogenous Nurr1 in PC12 cells and ectopically expressed Nurr1 in HeLa cells. (A) PC12 cells were treated with CHX in the absence or in the presence of Lactacystin (Lacta) for the times indicated and cell extracts analyzed by immunoblot with anti-Nurr1 antibodies. (B, D and F) HeLa cells were transiently transfected with full-length Nurr1, N-terminal flag-tagged Nurr1 or Nurr1 1–337 as indicated, after transfection cells were treated with CHX in the absence or in the presence of Lactacystin (Lacta) for the times indicated and cell extracts analyzed by immunoblot with anti-Nurr1 antibodies (B and D). Protein loading control was assessed by immunobloting with anti-tubulin antibodies. (C and E) Graphs show the quantification of immunoblots, and results are expressed as means ± s. e. m. from three different experiments of the indicated Nurr1 protein constructs. F, transfected cells were analyzed by indirect immunofluorescence with anti-Nurr1 antibodies (red channel), counterstained for nuclei with DAPI (blue channel) and imaging by confocal microscopy for subcellular localization of Nurr1.

**Figure 2:**
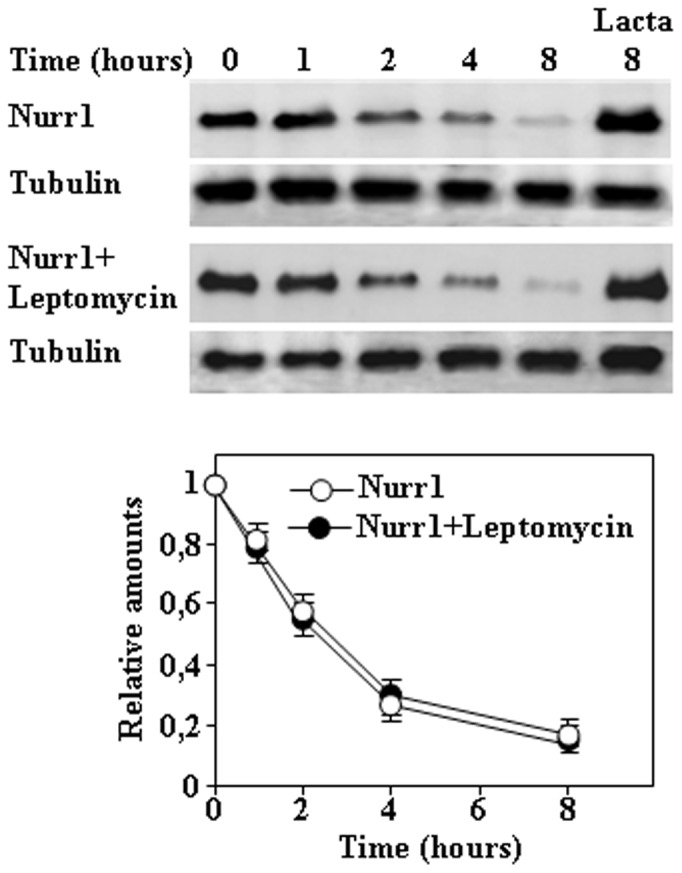
Effect of treatment of cells with Leptomycin B on the degradation of Nurr1. HeLa cells were transiently transfected with full-length Nurr1, after transfection cells were treated with CHX in the absence or in the presence of Lactacystin (Lacta) or Leptomycin B for the times indicated and cell extracts analyzed by immunoblot with anti-Nurr1 antibodies Protein loading control was assessed by immunobloting with anti-tubulin antibodies. Graphs show the quantification of immunoblots, and results are expressed as means ± s. e. m. from three different experiments.

To begin the study of the mechanism of Nurr1 degradation, we analyzed the degradation of endogenous Nurr1 in PC12 cells by treatment of cells with CHX. As shown in Fig. 1A and C, endogenous Nurr1 in PC12 cells is degraded with an apparent half-life of 3–4 h and the degradation was prevented by co-treatment with lactacystin, a specific and irreversible proteasome inhibitor. Next similar experiments were done by transfection of Nurr1 constructs in HeLa cells. Results presented in Fig. 1B and C shows that the half-life of transfected untagged Nurr1 shows similar kinetics as the endogenous Nurr1 in PC12 cells, and again the degradation was inhibited by co-treatment with lactacystin. To facilitate the study of the regions of Nurr1 that may be implicated in its degradation, we thought convenient to use a tagged version of Nurr1. We made a N-terminal flag-tagged version of Nurr1 that was transfected in HeLa cells and its half-life estimated by CHX treatment. As shown in Fig. 1B and C, the N-terminal flag-tagged Nurr1 has a longer half-life (12–14 h) than the untagged Nurr1 (3–4 h). All these results show that Nurr1 is degraded by the proteasome pathway with similar half-lives in cells of neuronal origin (PC12) and non-neuronal cells (HeLa) and suggested that the N-terminal region of Nurr1 may be important for degradation, because tagging Nurr1 at its N-terminal region produced an inhibition of its degradation rate in the cell. Accordingly, a N-terminal 1–337 Nurr1 construct was obtained. Results presented in Fig. 1D and E shows that a Nurr1 protein construct comprising aminoacids 1–337 was degraded with similar kinetics to the full length Nurr1. Note that all these Nurr1 constructs showed a nuclear localization by immunofluorescence confocal imaging (Fig. 1F), as the predicted nuclear localization signal of mouse Nurr1 is located within aminoacids 309-KRRRNR-314. Furthermore, inhibition of nuclear export by treatment of cells with Leptomycin B did not affect the rate of degradation of Nurr1 (Fig. 2). Taken together, these results indicate that the degradation of Nurr1 is mainly taken place in the cell nucleus.

### Delineation of the N-terminal region that targets Nurr1 for proteasomal degradation

**Figure 3:**
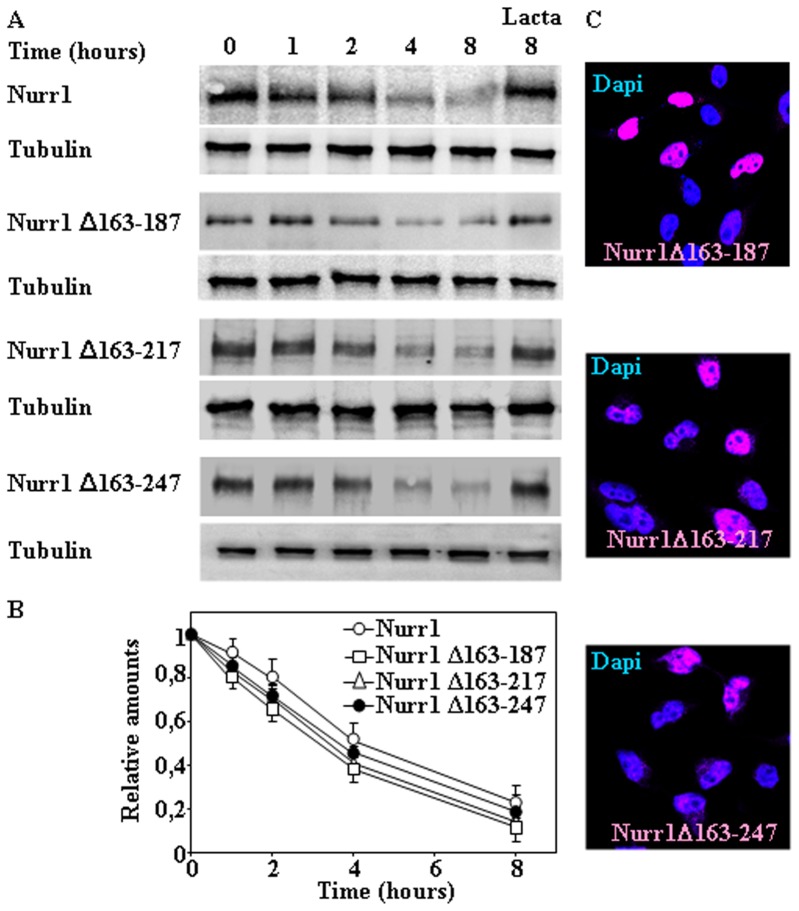
Effect of internal deletions within the N-terminal region of Nurr1 in its degradation. HeLa cells were transiently transfected with full-length Nurr1, Nurr1 Δ163–187, Δ163–217 and Δ163–249 as indicated, after transfection cells were treated with CHX in the absence or in the presence of Lactacystin (Lacta) for the times indicated and cell extracts analyzed by immunoblot with anti-Nurr1 antibodies (A). Protein loading control was assessed by immunobloting with anti-tubulin antibodies (A). (B) Graph shows the quantification of immunoblots, and results are expressed as means ± s. e. m. from three different experiments of the indicated Nurr1 protein constructs. (C) Cells were also analyzed by indirect immunofluorescence with anti-Nurr1 antibodies (red channel), counterstained for nuclei with DAPI (blue channel) and imaging by confocal microscopy for Nurr-1 subcellular localization.

**Figure 4:**
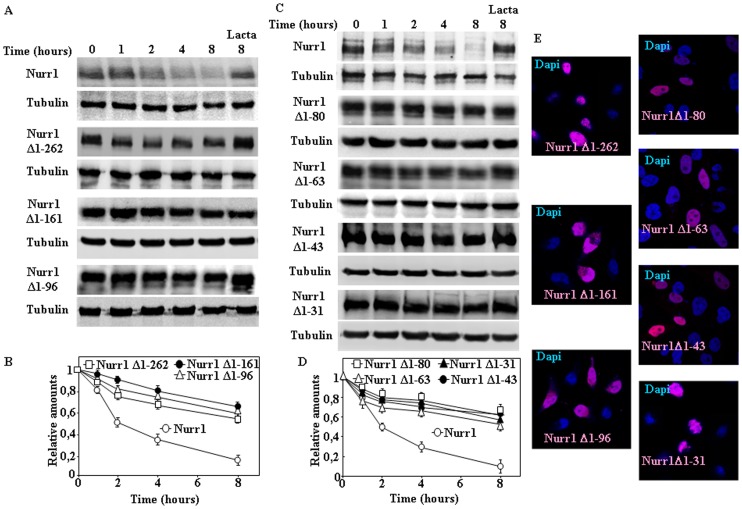
Effect of the deletions from the N-terminal of Nurr1 on its degradation. HeLa cells were transiently transfected with full-length Nurr1, Δ1–262, Δ1–161 and Δ1–96 (A) or Nurr1 Δ1–80, Δ1–63, Δ1–43, and Δ1–31 (C) as indicated, after transfection cells were treated with CHX in the absence or in the presence of Lactacystin (Lacta) for the times indicated and cell extracts analyzed by immunoblot with anti-Nurr1 antibodies (A and C). Protein loading control was assessed by immunobloting with anti-tubulin antibodies. (B and D) Graphs show the quantification of immunoblots, and results are expressed as means ± s. e. m. from three different experiments of the indicated Nurr1 protein constructs. (E) Cells were also analyzed by indirect immunofluorescence with anti-Nurr1 antibodies (red channel), counterstained for nuclei with DAPI (blue channel) and imaging by confocal microscopy for Nurr-1 subcellular localization of the different Nurr1 constructs as indicated.

The region of mouse Nurr1 from aminoacids 1–351 contains the AF-1 transactivation region of Nurr1, being the minimal transactivating region restricted to aminoacids 1–122 and the core transactivation sequence located between aminoacids 52–82 [16]. This region also contains the nuclear localization signal of Nurr1 309–314 as mentioned above, several phosphorylation sites (S126, T132 and T185) by MAPK/[16], [18] [20] and the region for the interaction with ERK1/2, ERK5 and LIMK1 that regulate Nurr1 transcriptional activity [21]. To define more precisely the sequence within the N-terminal region of Nurr1 required for targeting Nurr1 to proteasomal degradation, several deletion constructs were made from the convenient NdeI site of mouse Nurr1 corresponding to Met 162 and moving downstream, in all cases keeping the nuclear localization signal of Nurr-1. As shown in Fig. 3A and B, Nurr1 Δ163–187, Δ163–217, and Δ163–249 were degraded as efficiently as Nurr1 full-length in transfected cells, indicating that aminoacids 163–249 of the N-terminal region of Nurr1 did not seem to harbor the putative proteasomal targeting sequences of Nurr1, and as predicted those deletion constructs were located in the cell nucleus (Fig. 3C). Accordingly, we turned to the N-terminal portion and made three deletions Δ1–262, Δ1–161 and Δ1–96. Transfection of these N-terminal constructs into HeLa cells (Fig. 4A and B) showed that any of the deletions greatly diminished the degradation rate of Nurr1, being all those constructs also localized into the cell nucleus (Fig. 4E). These results clearly suggested that the region of Nurr1 spanning from aminoacid 1–96 contains the linear sequence within Nurr1 that is required for its proteasomal degradation. To further map the sequence within this N-terminal region, several deletion constructs from Met1 to 80, 63, 43, and 31 were generated. As shown in Fig. 4C and D all those deletions markedly reduced the degradation of the corresponding Nurr1 constructs, indicating that the minimal region required for efficient Nurr1 degradation seems to be present in the N-terminal region aminoacids 1–31 of the Nurr1 protein and again all of these deletion constructs localized in the cell nucleus (Fig. 4E).

### Ubiquitylation of Nurr1

**Figure 5:**
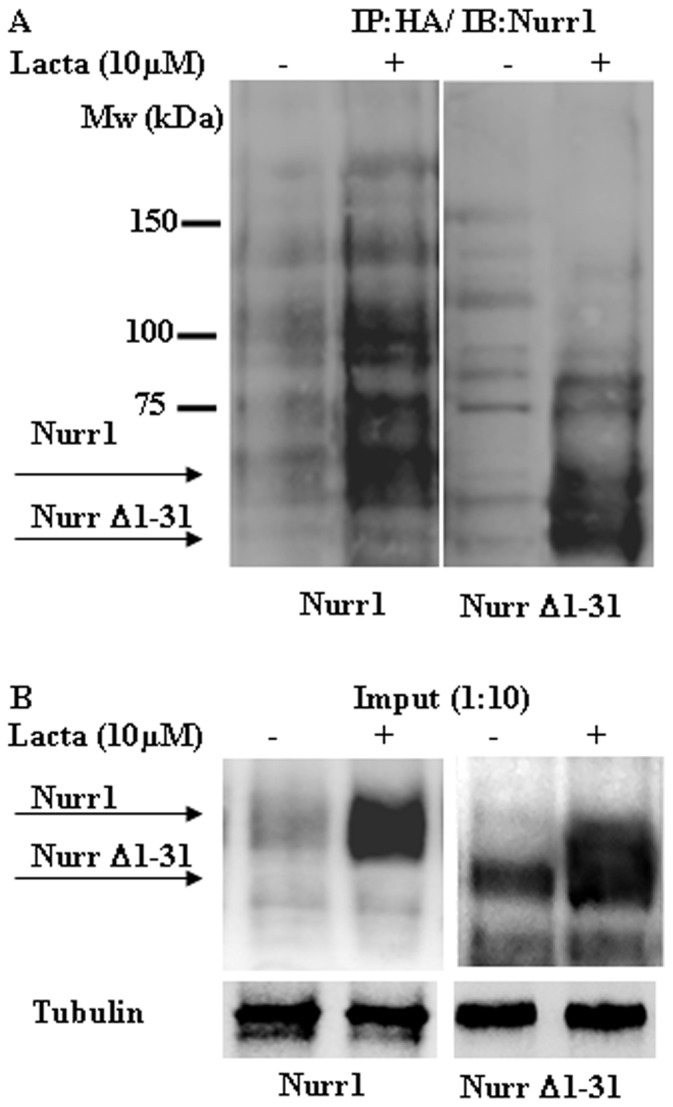
Ubquitylation of Nurr1 and Nurr1 Δ1–31. HeLa cells were co-transfected with Nurr1 full length or Δ1–31 and HA-ubiquitin and either untreated or treated with lactacystin (Lacta) as indicated, cell extracts were immunoprecipatated with anti-HA antibodies, analyzed by SDS-PAGE and immunoblotted with anti-Nurr1 antibodies (A). (B) Direct immunoblot with anti-Nurr1 antibodies of 1/10 of the amount of total cell extracts (shorter exposure than the upper panel) used for immunoprecipitation experiments shown in the upper panel. Protein loading control was assessed by immunobloting with anti-tubulin antibodies.

To check the ubiquitylation of Nurr1 and Nurr1 Δ1–31, HeLa cells were transiently co-transfected with those vectors and an HA-tagged ubiquitin expression vector. Ubiquitylated proteins were immunoprecipitated from solubilized cells using antibodies to the HA epitope and then separated by SDS-PAGE and immunoblotted with anti-Nurr1 antibodies. Results (Fig. 5A) showed that both Nurr1 and Nurr1 Δ1–31 are poly-ubiquitylated and those species accumulated when cells are treated with lactacystin. Note also that the steady state expression levels of Nurr1 Δ1–31 are higher than those of Nurr1 full length (Fig. 5B, input), as expected because of its longer half-life.

### Transcriptional transactivation and trans-repression by Nurr1 and N-terminal deletion mutants

**Figure 6:**
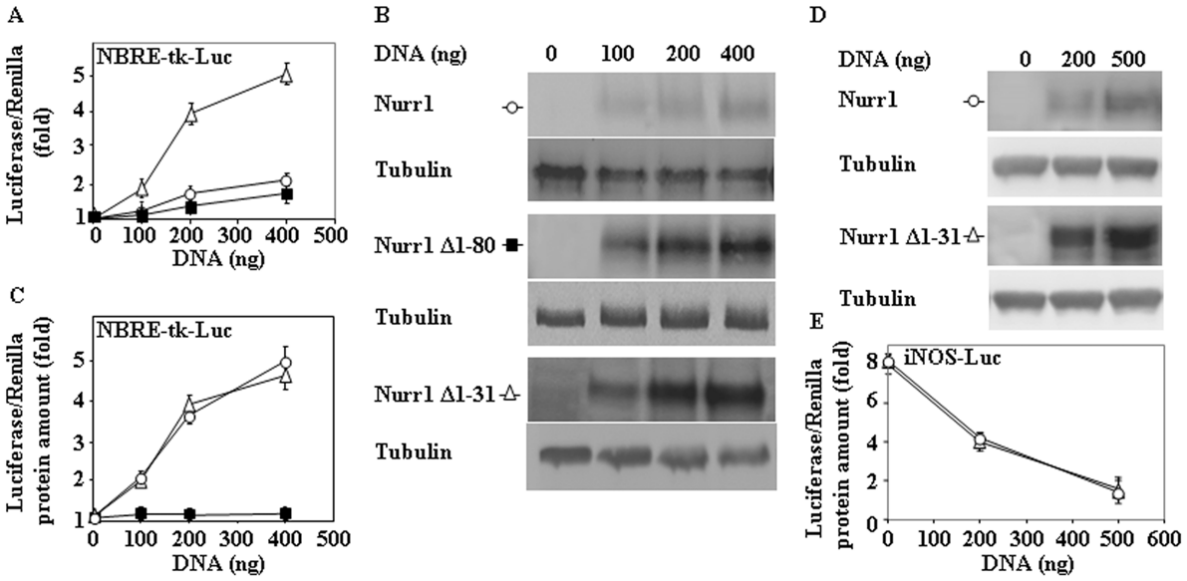
Transcriptional assays of Nurr1 and N-terminal deletion mutants. HeLa cells were cotransfected with empty pcDNA (0) or different doses of DNA of Nurr1 full length, Nurr1 Δ1–80 or Nurr1 Δ1–30 and constant amounts of DNA of NRBE firefly (400 ng) and *Renilla* luciferase (100 ng) reporters. (A) Graph showing the DNA dose response curve of the quotient of activities of firefly/*Renilla* luciferase (fold). (B) Immunoblot analysis with anti-Nurr1 antibodies of the DNA dose dependent expression of the different Nurr1 constructs transfected in HeLa cells, as indicated. (C) Graph showing the DNA dose response curve of the quotient of activities of firefly/*Renilla* luciferase divided by the amount of Nurr1 protein expression levels as judged by immunoblotting (fold). (D) RAW 264.7 cells were transfected with empty pcDNA (0), pcDNA Nurr1 or Nurr1 Δ1–31, and constant mounts of the reporter plasmid iNOS luciferase (400 ng) and Renilla (100 ng) as control. Cells were stimulated with bacterial LPS for 8 h and collected for Western immunblotting with anti-Nurr1 and anti-tubulin antibodies, as protein control loading (D) or for luciferase assays. (E) Graph shows the quotient of activities of firefly/*Renilla* luciferase divided by the amount of Nurr1 protein expression levels as judged by immunoblotting (fold).results are expressed as means ± s. e. m. from three different experiments.

Nurr1 binds the consensus NBRE site (AAAGGTCA) that is present in the promoter region of genes that are regulated by this nuclear receptor [9] [10] [11] [12]. As a consequence, we decided to study if the N-terminal Nurr1 deletion mutants can activate a 3xNBRE luciferase reporter construct using transient transfection assays. To that end, we performed co-transfections experiments of the NRBE firefly luciferase construct (with *Renilla* luciferase as a control) and different doses of Nurr1 full length and two deletion mutants Nurr1 Δ1–80 and Nurr1 Δ1–31. These experiments showed that different DNA concentrations of Nurr1 and Nurr1 Δ1–31 produced a dose-dependent activation of NBRE luciferase reporter (Fig. 6A), while Nurr1 Δ1–80 was defective in this transactivation assay. The degree of activation mirrored the increase observed in the amounts of Nurr1 protein levels (Fig. 6B). Accordingly, when the activation of the NRBE-luciferase reporter was corrected for the levels of expression of the respective Nurr1 proteins, the relative potency of Nurr1 full-length and Nurr1 Δ1–31 was not significantly different (Fig. 6C). In contrast, Nurr1 Δ1–80 that has also higher protein steady-state levels than Nurr1 full-length is completely ineffective in transactivation, as expected because this deletion mutant removes most of the AF-1 transactivation domain (aa 1–122) of Nurr1 [16].

Nurr-1 has also been described to act as a trans-repressor recruiting the CoREST corepressor complex that produces the inhibition of pro-inflamatory responses in astrocytes, microglia and macrophages [22]. Accordingly, it was interesting to study the behavior of Nurr1 Δ1–31 deletion mutant in this context. As shown in Fig. 6 D and E, the stimulation of an iNos luciferase reporter in RAW264.7 (a macrophage cell line) by LPS can be prevented by expression of Nurr1 as described previously [22], and the deletion mutant Δ1–31 has also similar effects as wild-type Nurr1. These results showed that deletion of the first 31 aminoacids of Nurr1 did not modify its ability to promote either transcriptional activation or trans-repression, behaving like Nurr1 full length, and its better performance can be explained by its decreased rate of degradation that resulted in an increase in the steady-state levels of the Nurr1 Δ1–31 respect to the full-length Nurr1.

## Discussion

The results presented in the present report show that the Nurr1 degradation is mediated by the ubiquitin-proteasome pathway, as it is inhibited by specific proteasome inhibitors and polyubiquitylated at its subcellular nuclear localization. The lysosomal pathway of degradation does not seem to be involved in Nurr1 degradation, as treatment of cells with leupeptin, E64b or chloroquine did not affect the steady-state levels of Nurr1 (data not shown). We have shown that one main determinant for proteasomal degradation of Nurr1 is located at the N-terminal region comprising aminoacids 1–31 that has no structural motives and scores very poorly as a possible PEST region [33]; and its deletion must not alter significantly its structure as it did not affect its localization in the nucleus or its function as a transcriptional regulator. Further experiments to precisely define the aminoacids responsible may not be an easy task because this N-terminal region may tolerate substantial aminoacid changes without affecting Nurr1 degradation. In fact, in an initial attempt to experimentally address this issue, we generated a triple point mutant where prolines (alpha-helix disrupter aminoacid) at positions 2, 12 and 17 were changed to Ala. This triple Pro/Ala Nurr1 mutant had a half-life not significantly different from the Nurr1 wild type (data not shown). Nevertheless, Nurr1 Δ1–31 is still poly-ubiquitylated and degraded while much less efficiently than Nurr1 wild type. The exact mechanism responsible of its reduced degradation rate remains to be determined. Nurr1 Δ1–31 may be either a less good substrate for the E3 ligase involved in the ubiquitylation of Nurr1 (decrease recognition or insufficient extension of the covalently bound poly-ubiquitin); and/or the poly-ubiquitylated Nurr1 Δ1–31 may be ineffectively recognized by the 19S proteasomal complex and/or ineffectively translocated to the catalytic chamber of the 20S proteasomal complex for degradation.

Nurr1 Δ1–31 is as potent as Nurr1 full-length in transactivation and trans-repression of luciferase reporter constructs, when corrected for the protein expression levels of both proteins, as Nurr1 Δ1–31 steady-state protein levels are higher than the levels of Nurr1 full length due to its longer half-life in the cell. These results are in agreement with the fact that the region 1–31 of Nurr1 is closed, but do not overlap, with the core region of the AF-1 transactivation domain of Nurr1, aminoacids 52–82 [16] and also will not interfere with ERK1/2 and ERK5 and LIMK1, as deletion of a.a 1–52 of Nurr11 does not affect the binding of any of these kinases that regulate Nurr1 activity [21]. The regions responsible of Nurr1 trans-repressor activity remains to be fully characterized. It is known that trans-repression of Nurr1 is suppressed by overexpression of Nurr1 DNA binding domain (DBD) indicating that the DBD is required for the interaction of Nurr1 with CoREST [22]. Certainly, the DBD of Nurr1 is not affected by the N-terminal 1–31 deletion of Nurr1, and as a consequence Nurr1 Δ1–31 behaves similar in trans-repression assays (shown here) as the wild type Nurr1.

The report that Nurr1 phosphorylation by AKT at Ser347 promotes its degradation [31] make us to repeat those experiments, and we found that the mutant Nurr1 S347A has the same half-life as the wild type Nurr1 (data not shown), consistently with the results presented here that the main determinant of Nurr1 degradation is located within aminoacids 1–31 of the N-terminal region of Nurr1.This apparent conflict of results may be due in part to the fact that Jo et al. [31] used for their studies a flag-tagged version of Nurr1. Tagging of Nurr1 at its N-terminus with a flag epitope, as shown here, resulted in an inhibition of its degradation rate in the cell by the ubiquitin-proteasome pathway (Fig. 1B), accordingly their results may not be relevant for the actual mechanism of degradation of the natural untagged Nurr1.

The presumptive role of Nurr1 in PD pathogenesis made us also to explore the possible effect of the mutation S125C that has been shown to be present in a PD patient [25] in Nurr1 degradation and consistently with the results presented here, the missense mutation that is located further downstream of the core N-terminal (aa1–31) did not affect the degradation rate of Nurr1 (data not shown), while this mutant has been reported to have a markedly reduced transactivation-activity [34]. A recent report also links Nurr1 with alpha-synuclein, a key protein in PD pathogenesis, and describes that alpha-synuclein over-expression by an unidentified mechanism promote Nurr1 degradation [35] linking mesencephalic Dopamine (mesDA) neurons maintenance and survival by Nurr1 and the expression levels of alpha-synuclein. We have studied the degradation of Nurr1 either by co-tranfection of alpha-synuclein and Nurr1 into HeLa cells, or by transfection of Nurr1 into N2a cells and N2a cells stably expressing alpha-synuclein [36] and we have found no effect of alpha-synuclein expression in the half-life of Nurr1 (3–4 h). In this context, recently published results using over-expression of alpha-synuclein in mesDA neurons also show a decrease in the expression of Nurr1 and Nurr1 downstream regulated genes, but this effect is mainly due to transcriptional down-regulation of Nurr1 by over-expression of alpha-synuclein [37].

The identification of the N-terminal region (a.a 1–31) in Nurr1 allowed us the production of a variant form of Nurr1 where that sequence was deleted and that was a rather stable protein in the cell and works very efficiently in promoting transactivation and trans-repression. Accordingly, the Nurr1 Δ1–31 becomes an ideal candidate to be used for direct gene transfer or transduction in cell-based therapies for the treatment of PD patients, because this construct keeps the main mechanisms of regulation of Nurr1 transactivation [18] [16] [19] [20] [21] and trans-repression known to be involved in tis anti-inflammatory properties [22], but it has a longer half-life than natural Nurr1: Nevertheless, it may be necessary to manipulate the expression levels of Nurr1 in neural stem/progenitor cells to mimic its expression during development of midbrain dopamine neurons in order to obtain good yields of neuronal DA cells for transplantation [38].

## References

[1] Law SW, Conneely OM, DeMayo FJ, O'Malley BW (1992) Identification of a new brain-specific transcription factor, NURR1. Mol Endocrinol 6: 2129–2135. 1491694 10.1210/mend.6.12.1491694

[2] Mages HW, Rilke O, Bravo R, Senger G, Kroczek RA (1994) NOT, a human immediate-early response gene closely related to the steroid/thyroid hormone receptor NAK1/TR3. Mol Endocrinol 8: 1583–1591. 7877627 10.1210/mend.8.11.7877627

[3] Zetterstrom RH, Williams R, Perlmann T, Olson L (1996) Cellular expression of the immediate early transcription factors Nurr1 and NGFI-B suggests a gene regulatory role in several brain regions including the nigrostriatal dopamine system. Brain Res Mol Brain Res 41: 111–120. 8883941 10.1016/0169-328x(96)00074-5

[4] Saucedo-Cardenas O, Conneely OM (1996) Comparative distribution of NURR1 and NUR77 nuclear receptors in the mouse central nervous system. J Mol Neurosci 7: 51–63. 8835782 10.1007/BF02736848

[5] Wallen A, Zetterstrom RH, Solomin L, Arvidsson M, Olson L, et al (1999) Fate of mesencephalic AHD2-expressing dopamine progenitor cells in NURR1 mutant mice. Exp Cell Res 253: 737–746. 10585298 10.1006/excr.1999.4691

[6] Saucedo-Cardenas O, Quintana-Hau JD, Le WD, Smidt MP, Cox JJ, et al (1998) Nurr1 is essential for the induction of the dopaminergic phenotype and the survival of ventral mesencephalic late dopaminergic precursor neurons. Proc Natl Acad Sci U S A 95: 4013–4018. 9520484 10.1073/pnas.95.7.4013 PMC19954

[7] Witta J, Baffi JS, Palkovits M, Mezey E, Castillo SO, et al (2000) Nigrostriatal innervation is preserved in Nurr1-null mice, although dopaminergic neuron precursors are arrested from terminal differentiation. Brain Res Mol Brain Res 84: 67–78. 11113533 10.1016/s0169-328x(00)00211-4

[8] Eells JB, Lipska BK, Yeung SK, Misler JA, Nikodem VM (2002) Nurr1-null heterozygous mice have reduced mesolimbic and mesocortical dopamine levels and increased stress-induced locomotor activity. Behav Brain Res 136: 267–275. 12385813 10.1016/s0166-4328(02)00185-7

[9] Sakurada K, Ohshima-Sakurada M, Palmer TD, Gage FH (1999) Nurr1, an orphan nuclear receptor, is a transcriptional activator of endogenous tyrosine hydroxylase in neural progenitor cells derived from the adult brain. Development 126: 4017–4026. 10457011 10.1242/dev.126.18.4017

[10] Kessler MA, Yang M, Gollomp KL, Jin H, Iacovitti L (2003) The human tyrosine hydroxylase gene promoter. Brain Res Mol Brain Res 112: 8–23. 12670698 10.1016/s0169-328x(02)00694-0

[11] Kim KS, Kim CH, Hwang DY, Seo H, Chung S, et al (2003) Orphan nuclear receptor Nurr1 directly transactivates the promoter activity of the tyrosine hydroxylase gene in a cell-specific manner. J Neurochem 85: 622–634. 12694388 10.1046/j.1471-4159.2003.01671.x

[12] Smits SM, Ponnio T, Conneely OM, Burbach JP, Smidt MP (2003) Involvement of Nurr1 in specifying the neurotransmitter identity of ventral midbrain dopaminergic neurons. Eur J Neurosci 18: 1731–1738. 14622207 10.1046/j.1460-9568.2003.02885.x

[13] Kadkhodaei B, Ito T, Joodmardi E, Mattsson B, Rouillard C, et al (2009) Nurr1 is required for maintenance of maturing and adult midbrain dopamine neurons. J Neurosci 29: 15923–15932. 20016108 10.1523/JNEUROSCI.3910-09.2009 PMC6666174

[14] Castro DS, Arvidsson M, Bondesson BM, Perlmann T (1999) Activity of the Nurr1 carboxyl-terminal domain depends on cell type and integrity of the activation function 2. J Biol Chem 274: 37483–37490. 10601324 10.1074/jbc.274.52.37483

[15] Wang Z, Benoit G, Liu J, Prasad S, Aarnisalo P, Liu X, et al (2003) Structure and function of Nurr1 identifies a class of ligand-independent nuclear receptors. Nature 423: 555–560. 12774125 10.1038/nature01645

[16] Nordzell M, Aarnisalo P, Benoit G, Castro DS, Perlmann T (2004) Defining an N-terminal activation domain of the orphan nuclear receptor Nurr1. Biochem Biophys Res Commun 313: 205–211. 14672718 10.1016/j.bbrc.2003.11.079

[17] Martinez-Gonzalez J, Badimon L (2005) The NR4A subfamily of nuclear receptors: new early genes regulated by growth factors in vascular cells. Cardiovasc Res 65: 609–618. 15664387 10.1016/j.cardiores.2004.10.002

[18] Kovalovsky D, Refojo D, Liberman AC, Hochbaum D, Pereda MP, et al (2002) Activation and induction of NUR77/NURR1 in corticotrophs by CRH/cAMP: involvement of calcium, protein kinase A, and MAPK pathways. Mol Endocrinol 16: 1638–1651. 12089357 10.1210/mend.16.7.0863

[19] Kim SJ, Kim JS, Cho HS, Lee HJ, Kim SY, et al (2006) Carnosol, a component of rosemary (Rosmarinus officinalis L.) protects nigral dopaminergic neuronal cells. Neuroreport 17: 1729–1733. 17047462 10.1097/01.wnr.0000239951.14954.10

[20] Zhang T, Jia N, Fei E, Wang P, Liao Z, et al (2007) Nurr1 is phosphorylated by ERK2 in vitro and its phosphorylation upregulates tyrosine hydroxylase expression in SH-SY5Y cells. Neurosci Lett 423: 118–122. 17681692 10.1016/j.neulet.2007.06.041

[21] Sacchetti P, Carpentier R, Segard P, Olive-Cren C, Lefebvre P (2006) Multiple signaling pathways regulate the transcriptional activity of the orphan nuclear receptor NURR1. Nucleic Acids Res 34: 5515–5527. 17020917 10.1093/nar/gkl712 PMC1636490

[22] Saijo K, Winner B, Carson CT, Collier JG, Boyer L, et al (2009) A Nurr1/CoREST pathway in microglia and astrocytes protects dopaminergic neurons from inflammation-induced death. Cell 137: 47–59. 19345186 10.1016/j.cell.2009.01.038 PMC2754279

[23] Le WD, Xu P, Jankovic J, Jiang H, Appel SH, et al (2003) Mutations in NR4A2 associated with familial Parkinson disease. Nat Genet 33: 85–89. 12496759 10.1038/ng1066

[24] Xu PY, Liang R, Jankovic J, Hunter C, Zeng YX, et al (2002) Association of homozygous 7048G7049 variant in the intron six of Nurr1 gene with Parkinson's disease. Neurology 58: 881–884. 11914402 10.1212/wnl.58.6.881

[25] Grimes DA, Han F, Panisset M, Racacho L, Xiao F, et al (2006) Translated mutation in the Nurr1 gene as a cause for Parkinson's disease. Mov Disord 21: 906–909. 16532445 10.1002/mds.20820

[26] Sleiman PM, Healy DG, Muqit MM, Yang YX, Van Der BM, et al (2009) Characterisation of a novel NR4A2 mutation in Parkinson's disease brain. Neurosci Lett 457: 75–79. 19429166 10.1016/j.neulet.2009.03.021 PMC4763922

[27] Chu Y, Le W, Kompoliti K, Jankovic J, Mufson EJ, Kordower JH (2006) Nurr1 in Parkinson's disease and related disorders. J Comp Neurol 494: 495–514. 16320253 10.1002/cne.20828 PMC2564615

[28] Le W, Pan T, Huang M, Xu P, Xie W, et al (2008) Decreased NURR1 gene expression in patients with Parkinson's disease. J Neurol Sci 273: 29–33. 18684475 10.1016/j.jns.2008.06.007 PMC2572302

[29] Jankovic J, Chen S, Le WD (2005) The role of Nurr1 in the development of dopaminergic neurons and Parkinson's disease. Prog Neurobiol 77: 128–138. 16243425 10.1016/j.pneurobio.2005.09.001

[30] Jorgensen JR, Juliusson B, Henriksen KF, Hansen C, Knudsen S, et al (2006) Identification of novel genes regulated in the developing human ventral mesencephalon. Exp Neurol 198: 427–437. 16473350 10.1016/j.expneurol.2005.12.023

[31] Jo AY, Kim MY, Lee HS, Rhee YH, Lee JE, et al (2009) Generation of dopamine neurons with improved cell survival and phenotype maintenance using a degradation-resistant nurr1 mutant. Stem Cells 27: 2238–2246 10.1002/stem.146 [doi]. 19522012 10.1002/stem.146 PMC2816355

[32] Vila-del SV, Diaz-Munoz MD, Fresno M (2007) Requirement of tumor necrosis factor alpha and nuclear factor-kappaB in the induction by IFN-gamma of inducible nitric oxide synthase in macrophages. J Leukoc Biol 81: 272–283 jlb.0905529 [pii];10.1189/jlb.0905529 [doi]. 17035338 10.1189/jlb.0905529

[33] Rice P, Longden I, Bleasby A (2000) EMBOSS: the European Molecular Biology Open Software Suite. Trends Genet 16: 276–277 S0168-9525(00)02024-2 [pii]. 10827456 10.1016/s0168-9525(00)02024-2

[34] Jacobsen KX, MacDonald H, Lemonde S, Daigle M, Grimes DA, et al (2008) A Nurr1 point mutant, implicated in Parkinson's disease, uncouples ERK1/2-dependent regulation of tyrosine hydroxylase transcription. Neurobiol Dis 29: 117–122. 17890097 10.1016/j.nbd.2007.08.003

[35] Lin X, Parisiadou L, Sgobio C, Liu G, Yu J, et al (2012) Conditional expression of Parkinson's disease-related mutant alpha-synuclein in the midbrain dopaminergic neurons causes progressive neurodegeneration and degradation of transcription factor nuclear receptor related 1. J Neurosci 32: 9248–9264 32/27/9248 [pii];10.1523/JNEUROSCI.1731-12.2012 [doi]. 22764233 10.1523/JNEUROSCI.1731-12.2012 PMC3417246

[36] Martin-Clemente B, Alvarez-Castelao B, Mayo I, Sierra AB, Diaz V, et al (2004) alpha-Synuclein expression levels do not significantly affect proteasome function and expression in mice and stably transfected PC12 cell lines. J Biol Chem 279: 52984–52990. 15466467 10.1074/jbc.M409028200

[37] Decressac M, Kadkhodaei B, Mattsson B, Laguna A, Perlmann T, et al (2012) α-Synuclein-Induced Down-Regulation of Nurr1 Disrupts GDNF Signaling in Nigral Dopamine Neurons. Science Translational Medicine 4: 163ra156. 10.1126/scitranslmed.3004676 23220632

[38] Park CH, Lim MS, Rhee YH, Yi SH, Kim BK, et al (2012) In vitro generation of mature dopamine neurons by decreasing and delaying the expression of exogenous Nurr1. Development 139: 2447–2451 dev.075978 [pii];10.1242/dev.075978 [doi]. 22627286 10.1242/dev.075978

